# The Comprehensive Analysis of Interferon-Related Prognostic Signature with regard to Immune Features in Ovarian Cancer

**DOI:** 10.1155/2022/7900785

**Published:** 2022-06-20

**Authors:** Yuanyuan An, Hua Duan

**Affiliations:** Gynecological Mini-Invasive Center, Beijing Obstetrics and Gynecology Hospital, Capital Medical University, Beijing Maternal and Child Health Care Hospital, 17 Qihelou Street, Dongcheng District, Beijing 100006, China

## Abstract

Interferon plays an important role in immune response of ovarian cancer. However, the expression pattern of interferon in ovarian cancer remains unclear. This study is aimed at exploring the expression profile of interferon-relate genes and constructing an interferon-based prognostic signature in ovarian cancer. The ovarian cancer samples collected from TCGA database were viewed as the training set, and ovarian cancer samples collected from GEO datasets were used as the independent validation sets. Univariate Cox regression analysis and multivariate Cox regression analysis were used to construct interferon-related signature, which worked as independent prognostic factor. Bioinformatics based on David software, GSEA, and R software were used to investigate the relationship between immune status and the signature in ovarian cancer. The signature showed close correlation with the status for ovarian cancer immune microenvironment, which might provide the possibility for clinical targeted therapy.

## 1. Introduction

As one of the common gynecological malignancies, the mortality of ovarian cancer still ranks first among gynecological tumors [1, 2]. Research showed that the 5-year survival rate of ovarian cancer patients was only 44% [3]. At present, the first-line treatment for ovarian cancer patients is still ovarian cancer reduction surgery combined with chemotherapy. Although tumor immunotherapy is in full swing, there is no drug targeting ovarian cancer immunotherapy for clinical application [4]. One of the main reasons is that ovarian cancer belongs to the “cold tumor,” which has poor response to immunotherapy. In fact, most of the prognostic-related signature researches for cancer patients are in view of entire transcriptome without considering the effect of biological process, which cannot reflect the immune status of cancer. Interferon (IFN) is a kind of cytokines secreted by host cells, which can participate in immune response, especially in cancer [5–7].

Nowadays, the researches on immunotherapy are very promising, such as PD-1/PD-L1 inhibitors and CAR-T therapy [8, 9]. Although the tumor mutation burden (TMB) of ovarian cancer is high, it still belongs to the category of “cold tumor,” which means deficiency of T lymphocyte infiltration in ovarian cancer immune microenvironment, resulting in not recognizing those tumor antigens [10]. Therefore, the benefit of immune checkpoint inhibitor treatment is less in ovarian cancer. At present, preclinical trials using modified T lymphocytes target the molecules including NY-ESO-1, HER2, MUC16, and p53 in ovarian cancer. In addition, the total response rate of ovarian cancer treated with anti-PD-1 antibody or anti-PD-L1 antibody alone was 10%~25% [11, 12]. Thus, it is important to find out new targets in immunotherapy in ovarian cancer, such as IFN-related genes.

As the first type of cytokines found, it was named “interferon” because the protein could interfere with virus replication. IFN can be divided into three types according to the receptors it binds. Each type of IFN can induce a specific immune response. In addition, IFN-mediated signaling promotes the upregulation of MHC-I and MHC-II and activates many downstream signal cascades to generate antiviral defense mechanisms. Studies also showed that IFN participated in the regulation of immune microenvironment in ovarian cancer. The CD8-positive lymphocytes secreted IFN-*γ*, which could upregulate PD-L1 in ovarian cancer cells, thus promoting the progression of tumor [13]. Gao et al. showed that IFN-*γ* could inhibit the progression of ovarian cancer through upregulating SOCS1 to inhibit the phosphorylation of STAT3 and STAT5, which further inhibited the migration and invasion and promoted apoptosis of ovarian cancer cells [14]. In ovarian cancer mouse model, combination of IL-4 pseudomonas exotoxin with IFN-*α* and IFN-*γ* could promote antitumor effect, which activated the mediators of apoptosis [15]. However, there were no researches focused on studying the comprehensive role of IFN-related genes played in ovarian cancer as a prognostic signature.

In our study, we constructed an IFN-related prognostic signature, which could not only worked as an independent prognostic predicting factor but also guided clinicians to pay attention to the role of IFN-related genes played in regulating the progression of ovarian cancer. Considering the specific role of IFN played in cancer immunotherapy, we tried to investigate the relationship between the novel signature with immunotherapy response, especially the immune checkpoints and immune status in ovarian cancer.

## 2. Materials and Methods

### 2.1. Public Ovarian Cancer Datasets

All the 458 IFN-related genes were gathered from gene set enrichment analysis (GSEA) database, including 25 gene sets (Supplementary Table 1). The normalized gene expression profiles and clinical parameters of ovarian cancer patients from The Cancer Genome Atlas (TCGA) database were downloaded from Firehose for further study. The independent validation microarray ovarian cancer cohorts were downloaded from the Gene Expression Omnibus (GEO) database (accession number: GSE26193 and GSE51088) based on HGU133 Plus 2 platform.

### 2.2. Construction of the IFN-Related Prognostic Signature

The univariate and multivariate Cox regression analyses were analyzed to evaluate the overall survival and gene expression values of IFN-related genes. The prognostic *p* value < 0.01 was considered significantly in univariate Cox regression analysis for further study. The Akaike information criterion (AIC) method was used to construct the most appropriate model through multivariate Cox regression analysis and construct the six-gene prognostic-related signature as follows: risk score = (0.51391 × MED1 expression) + (0.11716 × CCL15 expression) + (0.91753 × AXL expression)–(0.14807 × FZD5 expression)–(0.69668 × SLC30A8 expression) + (0.73590 × POLR3H expression). The heat map was constructed to view the relationship between clinical parameters and signature-related genes. The mutation platform of the signature genes was analyzed by cBioPortal from the website. In order to investigate the six IFN-related gene expression levels in various cancer cell lines and in distinct ovarian cancer cell lines, the Cancer Cell Line Encyclopedia (CCLE) database was used.

### 2.3. Kaplan-Meier Plotters

To explore the prognostic value of the six signature-related genes, the overall survival (OS), progression-free survival (PFS), and postprogression survival (PPS) were analyzed by the online database called Kaplan-Meier (K-M) plotter.

### 2.4. Biological Process and Pathway Enrichment Analysis

The “limma” R package was performed to ascertain differentially expressed genes (DEGs) related to the signature value in ovarian cancer from TCGA database. The DEGs between the high-risk group and the low-risk group were collected. Genes with logFC > 1.5 or <−1.5 and *p* value < 0.05 were collected for further study. David software from online software was used to analyze the GO and KEGG analysis.

### 2.5. Immune Cell Infiltration Analysis

Cell type identification by estimating the relative subset of known RNA transcripts (CIBERSORT) algorithm is a deconvolution algorithm developed by Binder G, which can accurately convert the expression data of tissue genes into the expression data for analyzing the composition and abundance of different immune cells in tissues. After standardizing the data, we transformed the standardized data into the relative expression data of 22 immune cells in ovarian cancer by CIBERSORT algorithm; the samples with *p* value < 0.05 were selected for further study. Based on the risk scores, patients were separated into two groups. The estimated immune cell proportions based on the CIBERSORT algorithm of the two groups were represented by box plot. The immune checkpoint inhibitor-related genes were collected; its relationship with risk scores was analyzed and showed by heat map. In addition, in order to investigate the relationship between immune cells and signature-related gene expression, online tool TIMER 2.0 database was used.

### 2.6. GSEA

GSEA was performed to investigate the gene set enrichment analysis based on risk score of the signature in ovarian cancer. To justify statistical significance, normalized enrichment score (NES) and false discovery rate (FDR) were performed.

### 2.7. DNA Methylation Analysis of the Six Genes from IFN-Related Signature

To investigate the role that DNA methylation of the six genes played in ovarian cancer, we used Gene Set Cancer Analysis (GSCA) database to explore the correlation between DNA methylation status and mRNA expression levels in ovarian cancer. Moreover, the survival analysis based on DNA methylation of genes in ovarian cancer was explored using cBioPortal database and GSCA database.

### 2.8. miRNA Analysis Based on the IFN-Related Genes

To investigate the potential miRNAs that correlated with the six IFN-related genes, we used the miRWalk database to investigate the potential miRNAs.

### 2.9. The Correlation between the Small Molecules or Drugs and the Signature using CMap Database

The CMap database was used to reflect the relationship among drugs, compounds, and diseases based on the alterations. Here, we used the CMap database to predict the potential small molecules or drugs targeting IFN-related signatures. Therefore, 87 genes upregulated in IFN-related signatures in ovarian cancer with poor prognosis were collected as candidate genes in the CMap database for analysis.

### 2.10. Statistical Analysis

In order to analyze the OS, disease-free survival (DFS), and PFS of ovarian cancer patients, Kaplan-Meier method and log-rank test were used. Univariate Cox proportional hazards regression and multivariate Cox proportional hazards regression were used to construct the model and analyze whether the gene or the signature could work as the prognostic factor of ovarian cancer patients independently. For the convenient analysis of clinical doctors, the prognostic nomogram was constructed through R software using rms package, and a calibration plot was created to perform the accuracy of nomogram. The relative scores among six genes were analyzed by Pearson analysis. The Student *t*-test was used to analyze the correlation between clinical parameters and risk scores, the analysis was two-tailed, and *p* value < 0.05 was viewed as statistically significant. All these data was analyzed using GraphPad Prism 7 software.

## 3. Results

### 3.1. Construction of an IFN-Related Prognostic Signature for Ovarian Cancer

**Figure 1:**
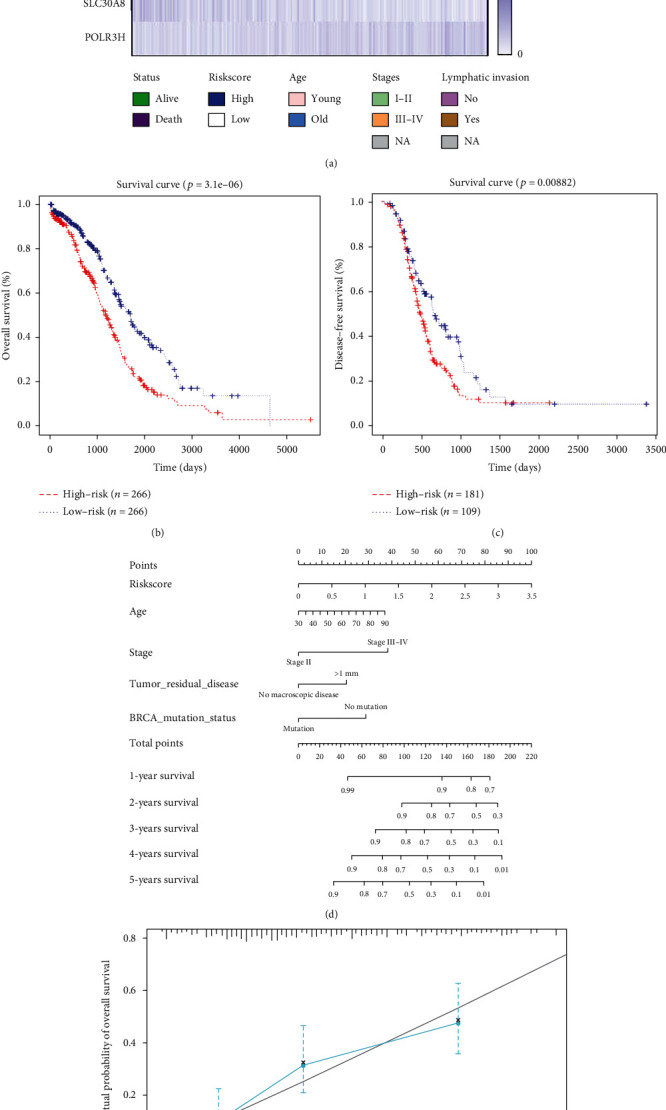
Relationship between IFN-related signature and prognosis in ovarian cancer. (a) The heat map analysis of the six-gene signature with clinical parameters. (b) The OS analysis based on the signature in TCGA database, the red line represented the high-risk group and the blue line represented the low-risk group. (c) The DFS analysis based on the signature in TCGA database, the red line represented the high-risk group and the blue line represented the low-risk group. (d) Construction of nomogram based on the signature and clinical parameters. (e) Calibration plot of the nomogram based on 5-year survival represented optimal agreement with the ideal model.

**Figure 2:**
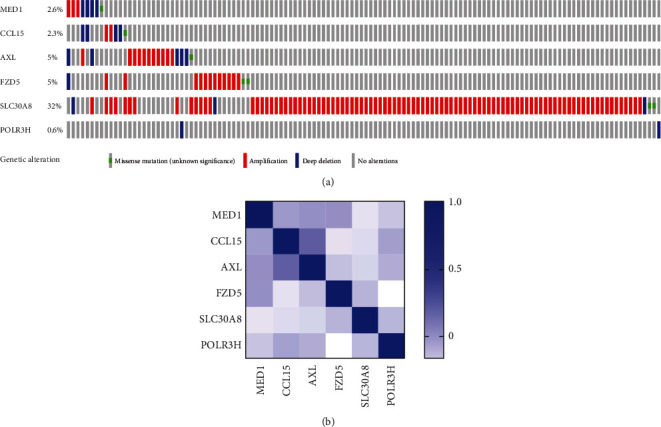
Mutation analysis of the six signature-related genes. (a) The mutation rate analysis of the signature-related genes in ovarian cancer through cBioPortal database, distinct genetic alterations were represented by various colors. (b) The correlation of the six genes based on Pearson analysis.

**Figure 3:**
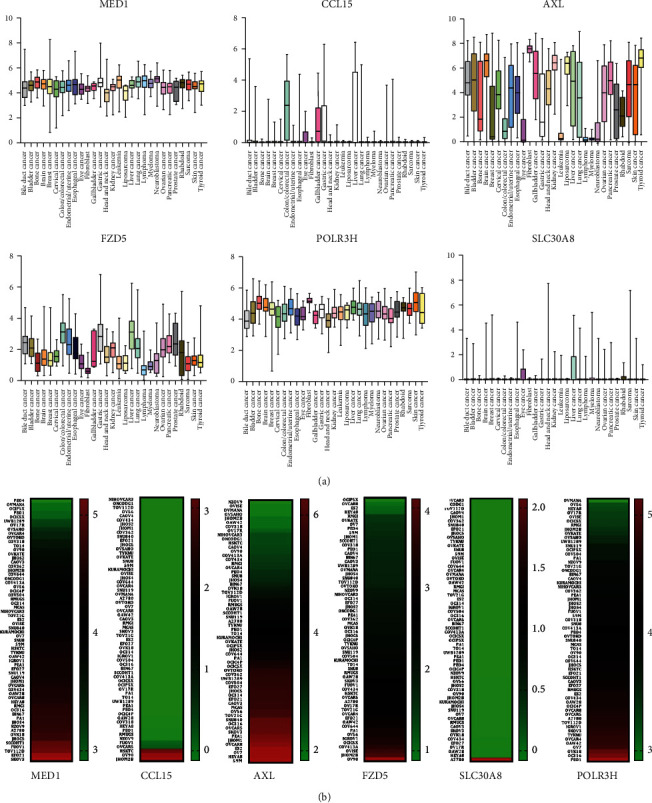
The expressions of the six signature-related genes in ovarian cancer cells based on the CCLE database. (a) The expression values of the six genes in various cancer cell lines in total. (b) The expression values of the six genes in distinct ovarian cancer cell lines, the red represented high expression of genes in cell lines and the green represented low expression of genes in cell lines.

458 IFN-related genes were gathered from the GSEA database for univariate Cox regression analysis, and seven genes (MED1, CCL15, AXL, TLR2, FZD5, SLC30A8, and POLR3H) with *p* value < 0.01 were collected (Table 1). In order to construct an IFN-related signature, multivariate Cox regression analysis was used, and six genes (MED1, CCL15, AXL, FZD5, SLC30A8, and POLR3H) with *p* value < 0.05 were included (Table 2). To investigate the correlation of the six genes with the clinical parameters in ovarian cancer, a heat map was constructed in Figure 1(a). Ovarian cancer patients were separated into two groups due to the expression of risk scores calculated by the formula above, and the OS and DFS were analyzed (Figures 1(b) and 1(c)). The results showed that patients with higher expressions of risk scores showed worse prognosis, both in OS and DFS. For the convenience of clinical doctors, a nomogram was constructed to estimate 5-year survival probability of ovarian cancer patients; the results showed that the risk scores played an important role in predicting the prognosis (Figure 1(d)). A calibration plot was constructed to show the optimal agreement for patients with 5-year survival, which represented the nomogram with good predictability (Figure 1(e)). According to the cBioPortal database, we represented the gene mutation rate in Figure 2(a), and the correlations between the six genes were analyzed by Pearson analysis (Figure 2(b)). In pan-cancer cell lines, the expression of the six IFN-related genes was relatively high in ovarian cancer cell lines among all the cancer types, which meant these genes played an important role in regulating ovarian cancer (Figure 3(a)). In ovarian cancer cell lines, the expression of the six IFN-related genes was quite different in distinct cell lines. What is important, we found that the expression of CCL15 and FZD5 was relatively low expressed in ovarian cancer cell lines, which also showed relative small proportion weight in the signature (Figure 3(b)).

### 3.2. The Signature Worked as an Independent Prognostic Factor for Ovarian Cancer

**Figure 4:**
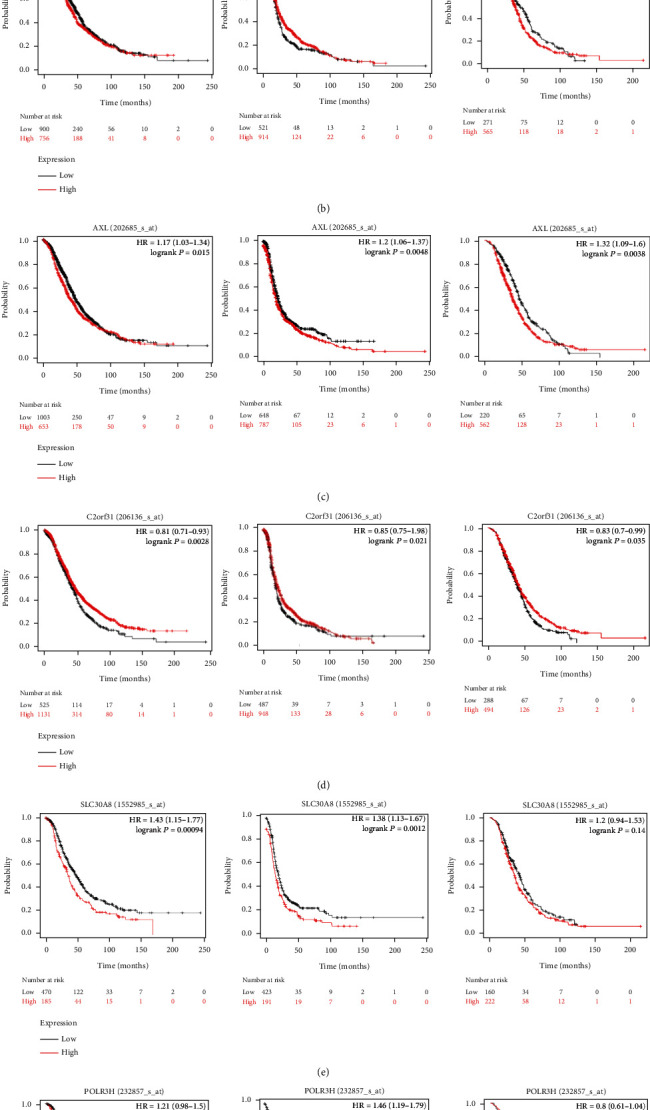
The survival analysis of six genes based on the K-M plotter database. Overall survival, progression-free survival, and postprogression survival analysis of (a) MED1, (b) CCL15, (c) AXL, (d) FZD5, (e) SLC30A8, and (f) POLR3H. The red line represented the high-expression group and the black line represented the low-expression group in ovarian cancer.

**Figure 5:**
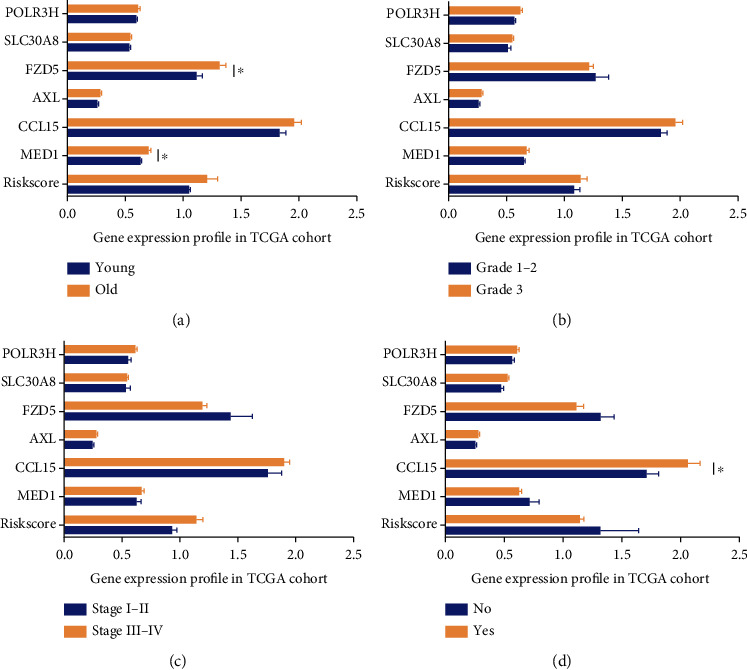
The correlation analysis of the signature and those related genes with the clinical parameters in ovarian cancer. The expression analysis of signature and genes based on (a) age, (b) grade, (c) stage, and (d) lymphatic invasion. ^∗^*p* < 0.05.

**Figure 6:**
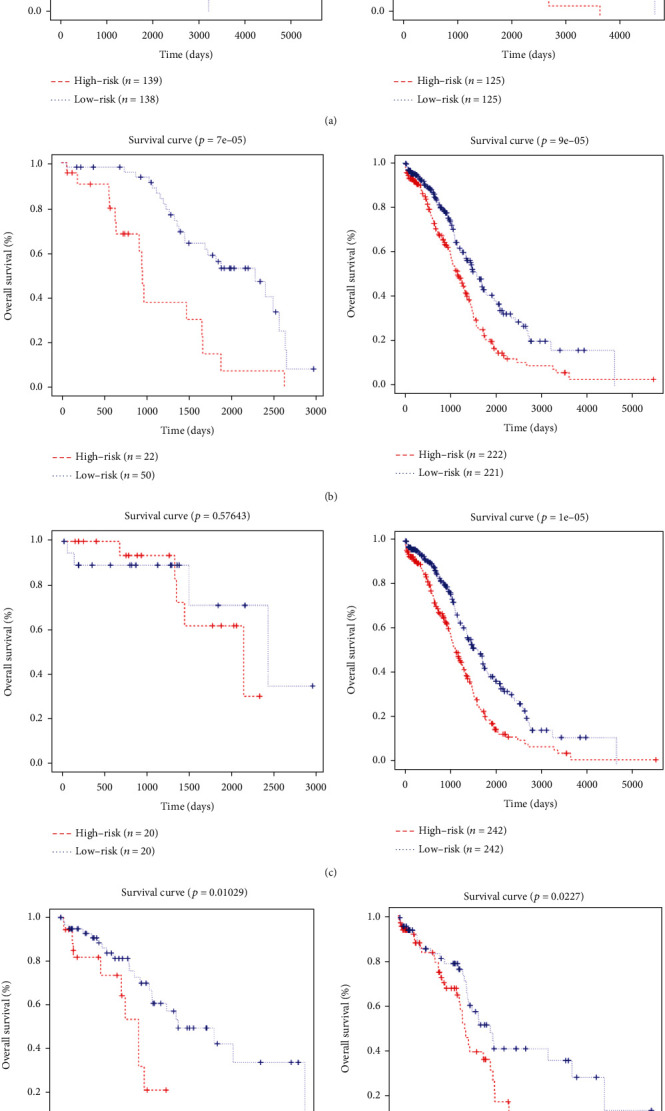
The stratified OS analysis of signature based on distinct clinical parameters. The OS of stratified analysis based on (a) age (young age and old age), (b) grade (early grade and late grade), (c) stage (early stage and late stage), and (d) lymphatic invasion (no lymphatic invasion and lymphatic invasion). The red line represented the high-risk group, and the blue line represented the low-risk group.

To investigate prognostic values of the six genes in ovarian cancer, the K-M plotter database was used to investigate the OS, PFS, and postprogression survival (PPS) of these genes. Results showed that the six genes all showed correlation with the prognosis of ovarian cancer patients (Figure 4). In order to investigate whether the signature worked as prognostic factor for predicting the prognosis of ovarian cancer independently, the multivariate Cox regression analysis was performed with the important clinical parameters, such as age, stage, grade, residual tumor size, BRCA1/2 mutation status, lymphatic invasion, and venous invasion of ovarian cancer patients. In Table 3, we found that the signature worked as an independent factor in predicting the OS of ovarian cancer. What is more, we found that the signature could also work as an independent factor for predicting the PFS of ovarian cancer in Table 4. To analyze the relationship of the signature and the six genes with those clinical parameters, including age, grade, stage, and lymphatic invasion, patients were divided based on the various clinical parameters (Figure 5). Thus, we found that the expression of FZD5 and MED1 correlated with the age, and CCL15 correlated with lymphatic invasion in ovarian cancer. To investigate the prognostic value of the signature in stratified cohorts, patients were divided into different groups according to age, stage, grade, and lymphatic invasion (Figure 6). The results showed that the signature could identify patients with prognosis precisely, without considering those clinicopathological parameters.

### 3.3. Validation of the Signature in the Independent Cohorts

**Figure 7:**
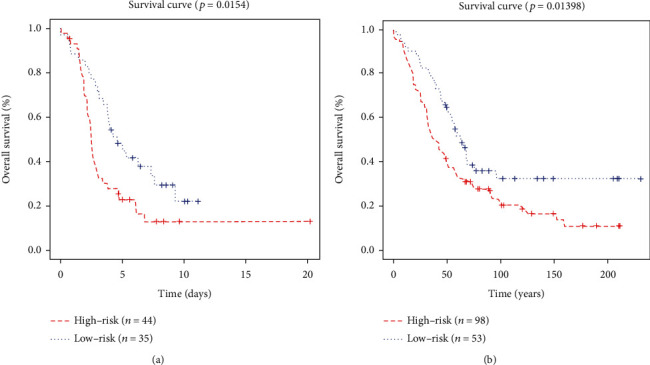
The OS analysis of the signature based on two independent cohorts from GEO database: (a) GSE26193 and (b) GSE51088. The red line represented the high-risk group, and the blue line represented the low-risk group.

In addition to TCGA cohorts, the signature was also validated in other independent cohorts from GEO dataset, including GSE26193 and GSE51088. In Figure 7, we analyzed the OS of the signature in these two datasets, which showed higher expression of risk scores with worse prognosis of ovarian cancer. For further validation of the role that the signature played in the independent datasets, univariate Cox regression and multivariate Cox regression survival analyses were used in GSE26193 and GSE51088, which showed that the signature could work as an independent prognostic factor for ovarian cancer patients (Supplementary Tables 2 and 3).

### 3.4. The Signature Correlated with Immune Response through GO and KEGG Analysis

**Figure 8:**
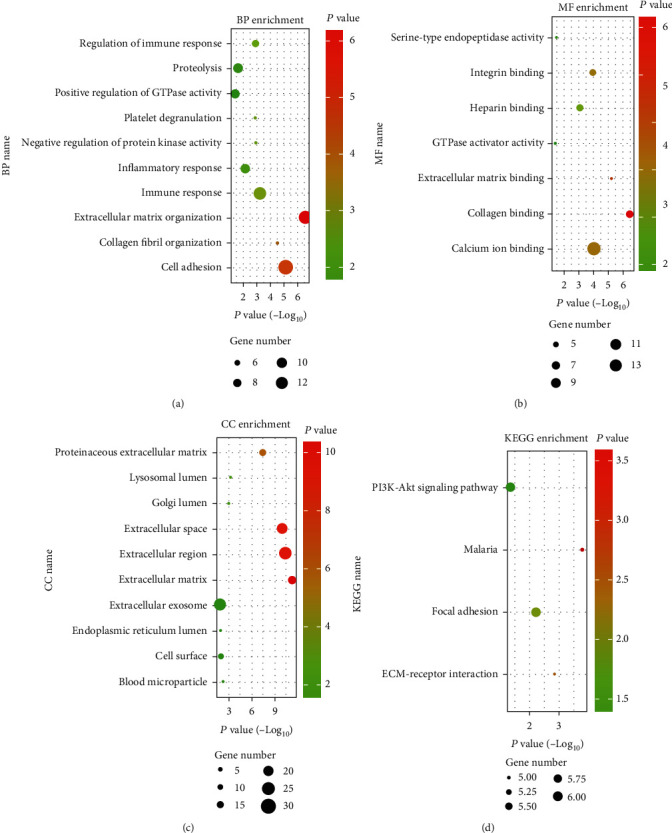
GO and KEGG analysis of the signature based on the DEGs of the risk score expressions: (a) BP enrichment; (b) MF enrichment; (c) CC enrichment; (d) KEGG enrichment. The color of the bubbles represented the *p* value of the signature, and the size of the bubbles represented the included gene numbers of the signature.

According to the risk scores, the patients were divided into two groups for further analysis. The DEGs between the high-risk group and the low-risk group were analyzed by “limma” packages from R software and showed in Supplementary Table 4. KEGG and GO analyses were used based on the 91 DEGs and showed the novel signature correlated with immune response and inflammatory response of ovarian cancer (Figure 8).

### 3.5. Immune Cell Infiltration Profile of the IFN-Related Signature

**Figure 9:**
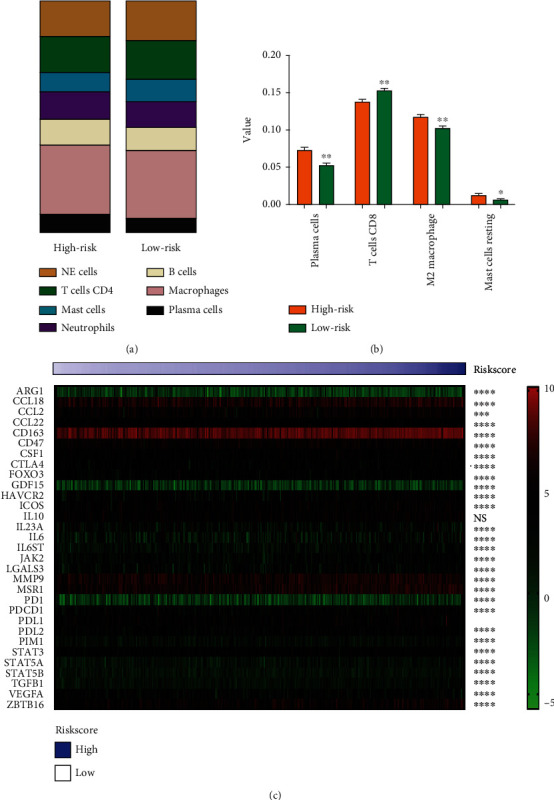
The immune analysis of the signature correlated with immune status of ovarian cancer. (a) Seven important immune cell expressions in the two groups. (b) Plasma cells, CD8+ T cells, M2 macrophages, and resting mast cells were significantly correlated with the signature. (c) Important immune checkpoint inhibitors were analyzed with risk score of the signature in ovarian cancer. ^∗^*p* < 0.05; ^∗∗^*p* < 0.01; ^∗∗∗^*p* < 0.001; ^∗∗∗∗^*p* < 0.0001; NS: not significant.

**Figure 10:**
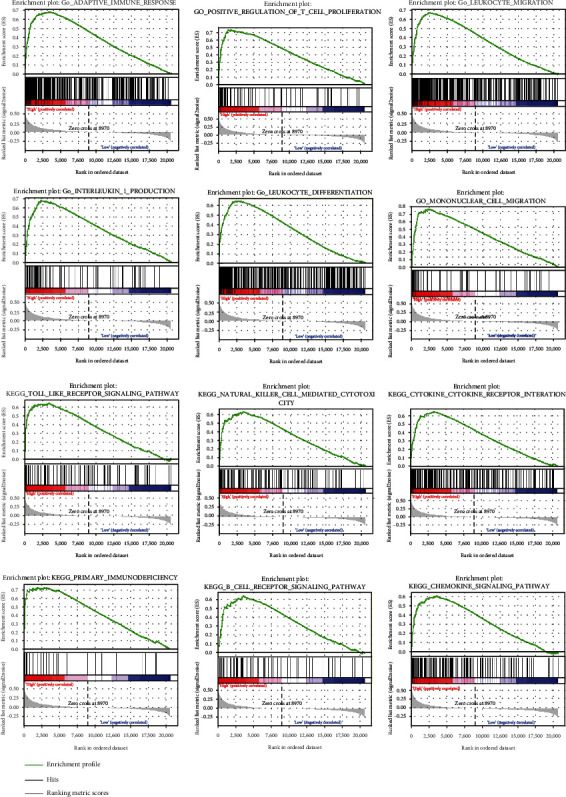
The GSEA was analyzed based on the expression of the signature in ovarian cancer.

**Figure 11:**
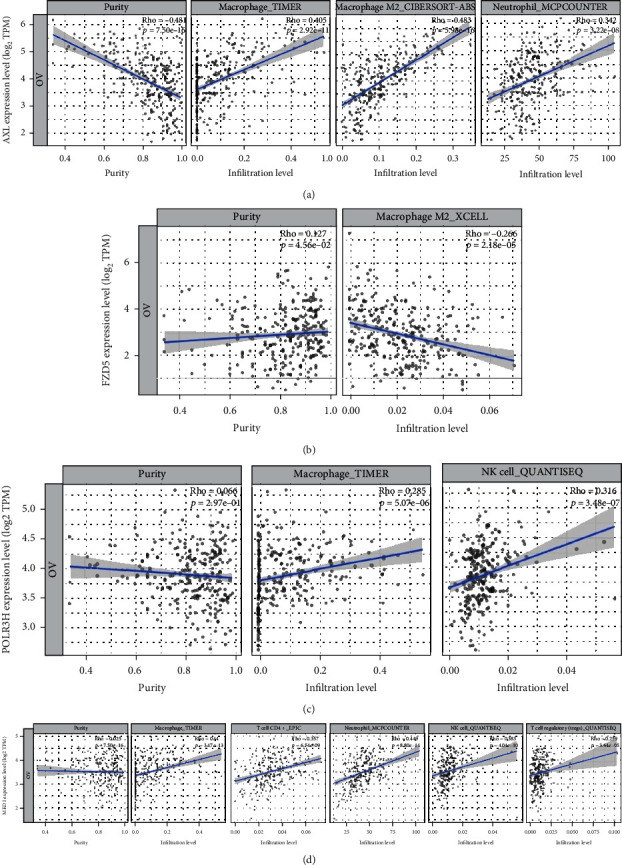
The six signature-related genes correlated with the immune cell expressions in ovarian cancer through TIMER 2.0 database. (a) AXL correlated with macrophages and neutrophils. (b) FZD5 correlated with M2 macrophages. (c) POLR3H correlated with macrophages and NK cells. (d) MED1 correlated with macrophages, neutrophils, NK cells, CD4^+^ T cells, and T cell regulatory (Tregs).

Due to the previous reports and the functional analysis of the signature, we further investigated the relationship between the signature and immune status of ovarian cancer.  Figure 9(a) shows that the 7 important immune cells represented different expression weights in the two groups based on the expression of the risk scores. For further analysis, the expression of plasma cells, M2 macrophage, and resting mast cells was significantly upregulated in the high-risk group, and CD8+ T cells was significantly upregulated in the low-risk group (Figure 9(b)). 31 molecules related with immune checkpoint inhibitors in cancer were collected, and most of the molecules showed significant correlation with the signature (Figure 9(c)). GSEA were analyzed based on the signature and showed the signature correlated with ovarian cancer immune microenvironment, including distinct biological functions of immune cells, such as B cells, T cells, and natural killer (NK) cells (Figure 10). The specific data of GSEA is shown in Table 5. To investigate the correlation between the six genes and immune cells, TIMER 2.0 database was used (Figure 11). The results showed that AXL correlated with macrophage and neutrophils, FZD5 correlated with M2 macrophages, and POLR3H correlated with macrophage and NK cells. The most important was that MED1 correlated with macrophages, neutrophils, NK cells, CD4^+^ T cells, and T cell regulatory (Tregs), which showed that it was closely related to the immune microenvironment in ovarian cancer.

### 3.6. DNA Methylation Analysis of the IFN-Related Genes in Ovarian Cancer

**Figure 12:**
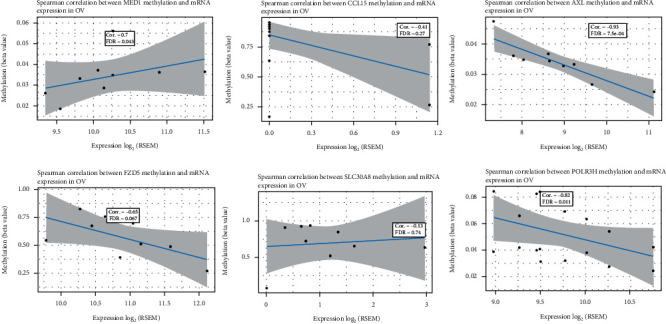
The correlation between gene methylation and mRNA expression levels in ovarian cancer through the GSCA database. The correlation scores and FDR were represented in the plots.

**Figure 13:**
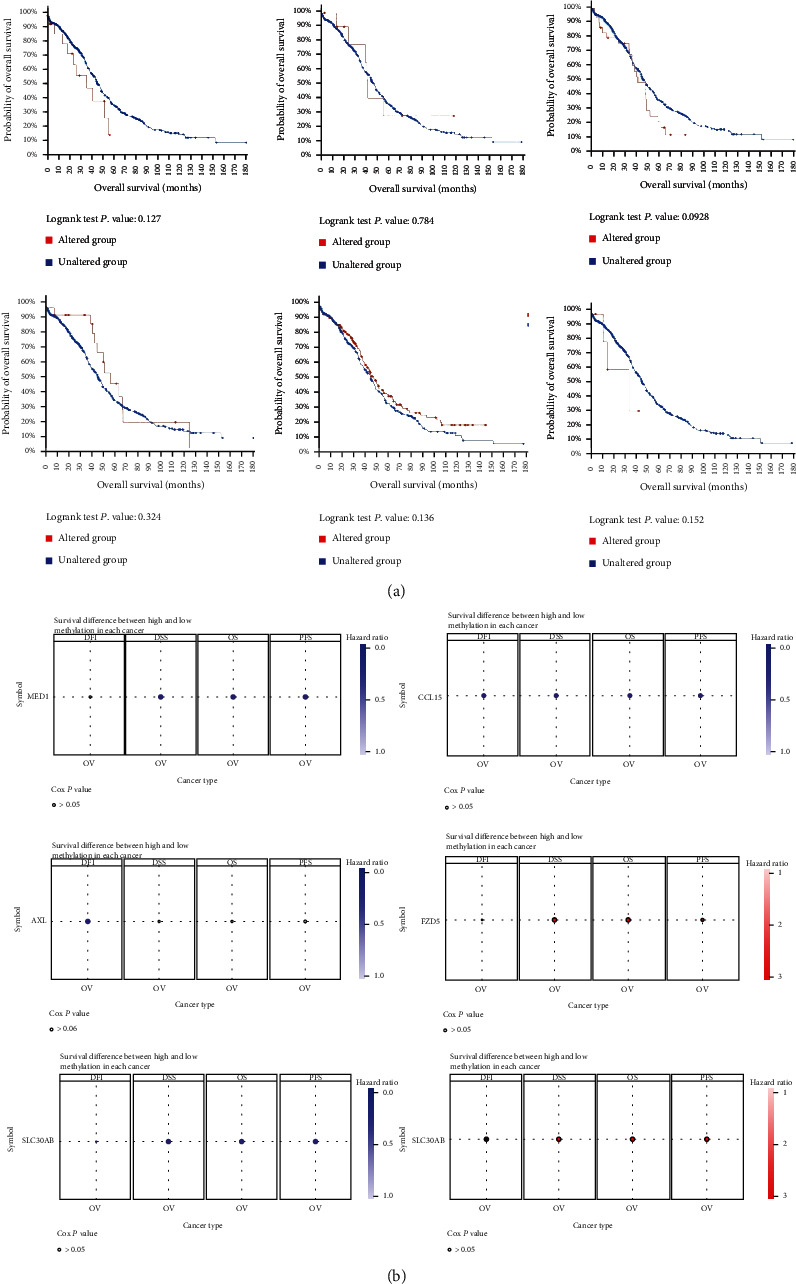
Survival analysis of methylated signature-related genes in ovarian cancer. (a) The OS analysis of six genes' (MED1, CCL15, AXL, FZD5, SLC30A8, and POLR3H) methylation in ovarian cancer using cBioPortal database, the red line represented the altered group and the blue line represented the unaltered group. (b) Total survival analysis (DFI, DSS, OS, and PFS) of six genes' methylation in ovarian cancer using the GSCA database.

To analyze the role that methylated signature-related genes played in ovarian cancer, we first explored the correlation between mRNA expression levels of the genes with its DNA methylation in Figure 12. The results showed that MED1 mRNA expression positively correlated with its methylation status, while AXL and POLR3H mRNA expression negatively correlated with its methylation status. To further investigate whether the methylated genes correlated with prognosis of ovarian cancer patients, OS analysis based on cBioPortal database is represented in Figure 13(a). The results showed that the six methylated genes could not influence the prognosis of ovarian cancer patients. Other survival analyses including disease-free interval (DFI), disease-specific survival (DSS), OS, and PFS of the methylated genes in ovarian cancer were investigated using the GSCA database in Figure 13(b). In Supplementary Table 5, the results showed the *p* value of survival analysis of the six methylated gene in ovarian cancer. However, there was no significant correlation between DNA methylation and prognosis value in ovarian cancer. Thus, these results suggested that these signature-related genes could regulate the prognosis of ovarian cancer patients through its mRNA expression values, not due to its DNA methylated status.

### 3.7. miRNA Analysis of the IFN-Related Genes

**Figure 14:**
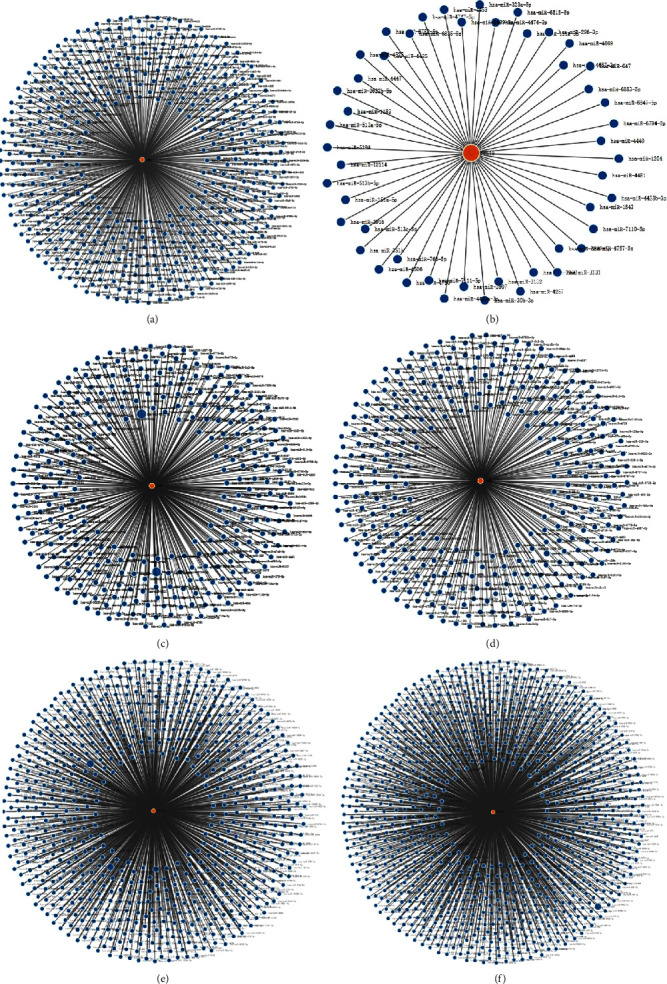
Analysis of the miRNA networks according to the signature-related genes in ovarian cancer using miRWalk database: (a) MED1; (b) CCL15; (c) AXL; (d) FZD5; (e) SLC30A8; (f) POLR3H.

In Figure 14, we analyzed the miRNA-regulated networks of the six IFN-related genes using miRWalk database. The results showed that the IFN-related genes correlated with multiple miRNAs, expect for CCL15.

### 3.8. Investigation of the Small Molecules or Drugs Targeting IFN-Related Signature in Ovarian Cancer

**Figure 15:**
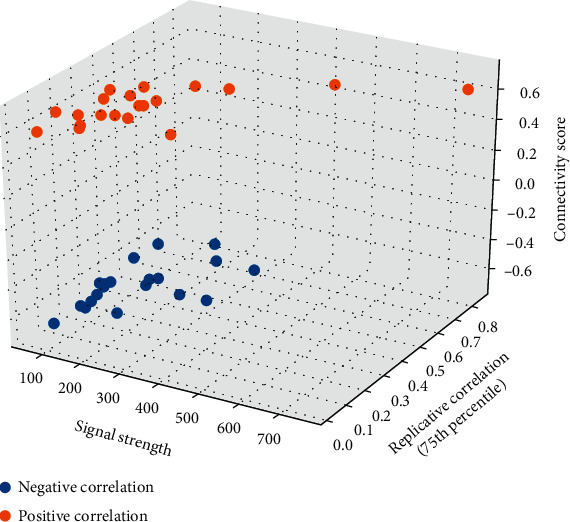
Three dimensional plots showed the correlation among signal strength, replicative correlation, and the connectivity score of the candidate agents, which were assessed by determining the differential genetic signature based on the risk scores of IFN-related signature. The positive connectivity scores represented the small molecules or drugs induced a similar signature to the IFN-related signature panel, while the negative connectivity scores represented the small molecules or drugs induced a signature that was opposite of the IFN-related signature panel.

The small molecules or drugs that might target the oncogenic pathways based on the expression of IFN-related signature in ovarian cancer were explored by the CMap database. Due to the risk scores of the IFN-related signature in ovarian cancer, DEGs were explored as above. Here, we found 87 genes significantly upregulated in the high-risk group, which was used for further analysis in the CMap database. The top 20 positive correlation drugs and top 20 negative correlation drugs were collected according to the connectivity scores (Figure 15). The results showed that the drugs of bromodomain inhibitor PFI-1 (connectivity score = −0.6848, TAS = 0.3792), sorafenib (connectivity score = −0.6523, TAS = 0.3490), ATM kinase inhibitor CGK-773 (connectivity score = −0.6488, TAS = 0.4499), and mineralocorticoid antagonist spironolactone (connectivity score = −0.6349, TAS = 0.3050) with the signature strength larger than 200 and replicative correlation larger than 0.2 could be viewed as the candidate drugs targeting IFN signature in ovarian cancer (Supplementary Table 6).

## 4. Discussion

Ovarian cancer is one of the most common malignancies in women with high mortality rate and poor prognosis. Nowadays, computational models have been constructed based on the sequencing analysis to explore the possible biomarkers in cancer. IFN is a kind of cytokine family with extensive biological activities produced by different cells, which function in antiviral, immune regulation, inhibition of cell proliferation, and so on. At present, type I interferon has been used in clinical treatment of hematological tumors and solid tumors [16–18]. Current studies showed that antitumor mechanisms of IFN mainly included inhibiting the activity of endothelial cells, which mediated tumor angiogenesis, and enhancing the immunogenicity of immune cells, including T cells, NK cells, DC cells, and macrophages, which showed the close relationship between IFN expression and immune status of cancer, thus influencing the prognosis of cancer patients [19–22]. Here, we first investigated the role that IFN-related genes played in ovarian cancer; thus, we constructed a novel IFN-related signature and investigated its role played in immunotherapy and prognosis of ovarian cancer.

There were six IFN-related genes (MED1, CCL15, AXL, FZD5, SLC30A8, and POLR3H) included in our signature. Some studies showed that these genes played an important role in the pathogenesis of ovarian cancer. In research of colorectal cancer and ovarian cancer, the mutation of MED1 associated with microsatellite instability of cancer cells, which promoted tumorigenesis [23]. CCL15 mainly correlated with phenotype of ovarian cancer, which showed significantly upregulated in mucinous ovarian cancer [24]. FZD5 worked as the receptor of WNT signaling. In HGSOC, Wnt7B-FZD5 signaling could regulate the epithelial phenotype and stem-like property [25]. In fact, Wnt5A-FZD5 signaling participated in regulating the adhesion of ovarian cancer cells. As for AXL, there were lots of researches focused on ovarian cancer, especially in chemoresistance. Inhibition AXL could promote the chemosensitivity to cancer cells, especially platinum and taxane in ovarian cancer [26]. In ovarian cancer, AXL could promote glycolysis mediated by phosphorylating PKM2 at Y105, thus inhibiting the chemoresistance of ovarian cancer cells to cisplatin [27]. However, no related researches of SLC30A8 and POLR3H have been studied in ovarian cancer. Although these genes showed the correlation with progression of ovarian cancer, there was no study that combined these genes as the biomarker for studying the role they played in influencing the prognosis of immune status of ovarian cancer.

As a promising therapeutic target in cancer, the role IFN played in cancer has been widely discussed. Researches showed that IFN could not only regulate the cancer cells but also influence cancer immune microenvironment, including cancer-associated fibroblasts and other immune cells [28–30]. In our study, we first explored the role IFN played in ovarian cancer through constructing a prognostic-related signature using IFN-related genes. The complex role IFN played in ovarian cancer has been widely discussed, and no conclusion has been reached yet. Therefore, we established an IFN-related signature as a new prognostic model, which divided patients into two groups, namely, high-risk group and low-risk group. The performance of the signature was verified by survival analysis and validated by two independent cohorts. The most important thing was that the signature worked as prognostic factor of ovarian cancer patients independently. For further validation, in the stratified analysis, we found that the signature could also correlate with the prognosis of ovarian cancer independent of those clinical parameters. As we all know, nomogram is a good method to predict the prognosis for ovarian cancer patients in clinic. Therefore, a nomogram was constructed including these important clinical parameters, with the risk scores of the signature, which showed that the risk scores of signature was very important in predicting the prognosis of ovarian cancer. Through all these validation methods, we found that the signature worked as the prognostic characteristics for ovarian cancer patients.

As early as in 1969, it was first reported that IFN could inhibit tumor growth in animals. In 1986, FDA approved IFN as the drug for antitumor therapy. However, in recent years, people began to rethink the important role of IFN in determining tumor development, disease progression, and treatment response. Studies showed that mesenchymal stem cells could promote tumor growth by releasing high concentrations of nitric oxide and recruiting macrophages to tumor sites [31, 32]. After genetic engineering, mesenchymal stem cells secreted IFN*α* which could play the antitumor role [33]. In our study, we found the signature-related genes such as AXL, FZD5, POLR3H, and MED1 were related to the immune cell expressions in ovarian cancer. Based on DEGs between these two groups according to the risk scores, GO and KEGG analysis combined with GSEA analysis was performed, which showed that immune status and inflammatory status of ovarian cancer were correlated with the IFN-related signature. In fact, the signature not only correlated with the immune cells expressions in ovarian cancer microenvironment but also correlated with different kinds of immune checkpoint inhibitors. Most importantly, we found that plasma cells, M2 macrophages, and resting mast cells were significantly upregulated in the high-risk group, which showed worse prognosis. At the same time, CD8+ T cells were upregulated in the low-risk group, which showed favorable prognosis. These results reflected the accuracy of our signature to a certain extent. Thus, we assumed that the novel IFN-related signature significantly related to the immune microenvironment status of ovarian cancer, which may be a new direction of immunotherapy for ovarian cancer.

## 5. Conclusion

In conclusion, we constructed a novel IFN-related prognostic signature associated with the immune status of ovarian cancer, validated by two other independent cohorts. Therefore, this signature could predict the prognosis for ovarian cancer, which also might be helpful for illustrating the application of immunotherapy for ovarian cancer patients.

## Figures and Tables

**Table 1:** The IFN-related genes with the prognostic *p* value < 0.01 using univariate Cox regression analysis.

Gene	HR	*z*	*p* value
MED1	1.723210145	3.608815486	0.000307598
CCL15	1.179089666	3.491151846	0.000480943
AXL	3.470834022	3.079644755	0.002072476
TLR2	1.021883244	2.817336898	0.00484237
FZD5	0.823927648	-2.750997775	0.005941405
SLC30A8	0.458055386	-2.625493506	0.008652346
POLR3H	2.227603436	2.609654178	0.009063379

**Table 2:** The IFN-related genes with the prognostic *p* value < 0.05 using multivariate Cox regression analysis.

Gene	Coef	*z*	*p* value
MED1	0.51391	3.415	0.000638
CCL15	0.11716	2.353	0.018634
AXL	0.91753	2.193	0.028311
FZD5	-0.14807	-2.068	0.038654
SLC30A8	-0.69668	-2.351	0.018701
POLR3H	0.73590	2.348	0.018898

**Table 3:** The OS using univariate and multivariate Cox regression analysis of the signature in TCGA database.

Variable	Overall survival			
	Univariate		Multivariate	
	HR	*p* value	HR	*p* value
The signature	0.570	<0.001	0.593	0.002
Age	1.019	<0.001	1.017	0.021
Grade	1.180	0.055		
Stage	1.659	0.003	1.726	0.064
Residual tumor size	0.437	<0.001	1.726	0.025
BRCA1/2 mutation	2.021	<0.001	2.030	0.001
Lymphatic invasion	1.422	0.114		
Venous invasion	0.973	0.917		

**Table 4:** The DFS using univariate and multivariate Cox regression analysis of the signature in TCGA database.

Variable	Disease-free survival			
	Univariate		Multivariate	
	HR	*p* value	HR	*p* value
The signature	0.671	0.009	0.589	0.002
Age	1.012	0.058		
Grade	1.219	0.051		
Stage	1.432	0.084		
Residual tumor size	0.478	0.002	0.485	0.005
BRCA1/2 mutation	1.752	0.020	1.862	0.004
Lymphatic invasion	1.491	0.148		
Venous invasion	0.714	0.311		

**Table 5:** The GSEA statistics data of the signature based on TCGA database.

Enrichment plot	ES	NES	FDR	FWER	Nominal *p* value
GO: adaptive immune response	0.6735596	2.3682117	0	0	0
GO: positive regulation of T cell proliferation	0.72983944	2.3148284	0	0	0
GO: leukocyte migration	0.6692668	2.3699362	0	0	0
GO: interleukin-1 production	0.7497201	2.3146412	0	0	0
GO: leukocyte differentiation	0.64995396	2.3203151	0	0	0
GO: mononuclear cell migration	0.7668914	2.3711784	0	0	0
KEGG: toll-like receptor signaling pathway	0.65039474	2.0473661	0	0	0
KEGG: natural killer cell-mediated cytotoxicity	0.6354623	2.0981436	0	0	0
KEGG: cytokine-cytokine receptor interaction	0.6487897	2.2508793	0	0	0
KEGG: primary immunodeficiency	0.7338493	1.9932709	0.0000798	0.001	0
KEGG: B cell receptor signaling pathway	0.6443853	1.9716772	0.000128	0.002	0
KEGG: chemokine signaling pathway	0.6134719	2.0801277	0	0	0

## Data Availability

All data obtained and/or analyzed during the current study were available from the corresponding authors on reasonable request.

## Supplementary Materials

Supplementary Materials Table 1: the candidate IFN-related genes. Supplementary Table 2: the univariate and multivariate Cox regression analysis of overall survival in GSE26193. Supplementary Table 3: the univariate and multivariate Cox regression analysis of overall survival in GSE51088. Supplementary Table 4: DEGs between two groups based on the risk scores of signature. Supplementary Table 5: survival analysis of DNA methylated signature-related genes using the GSCA database. Supplementary Table 6: prediction of top and bottom 20 drugs targeting positive and negative correlation using CMap database based on signature.
