# Supplementary material for: The Comprehensive Analysis of Interferon-Related Prognostic Signature with regard to Immune Features in Ovarian Cancer
Source: Dis Markers. 2022 Jun 20;2022:7900785. doi: 10.1155/2022/7900785 (PMC9236773; doi:10.1155/2022/7900785)
Supplement: Supplementary Materials — Table 1: the candidate IFN-related genes. Supplementary Table 2: the univariate and multivariate Cox regression analysis of overall survival in GSE26193. Supplementary Table 3: the univariate and multivariate Cox regression analysis of overall survival in GSE51088. Supplementary Table 4: DEGs between two groups based on the risk scores of signature. Supplementary Table 5: survival analysis of DNA methylated signature-related genes using the GSCA database. Supplementary Table 6: prediction of top and bottom 20 drugs targeting positive and negative correlation using CMap database based on signature. [file 7900785.f1.docx]

**Supplementary Table 1: The candidate IFN-related genes**

| ABCE1 | CCL4 | DDIT3 | HLA-DQA2 | IFNA7 | ISG20 | NLRX1 | PPARG | SLC26A6 | TRIM25 |
| --- | --- | --- | --- | --- | --- | --- | --- | --- | --- |
| ABL1 | CCL5 | DDX3X | HLA-DQB1 | IFNA8 | ISL1 | NMI | PPM1B | SLC30A8 | TRIM26 |
| ACOD1 | CCL7 | DDX41 | HLA-DQB2 | IFNAR1 | ITCH | NOS2 | PQBP1 | SLC7A5 | TRIM27 |
| ACTR2 | CCL8 | DDX58 | HLA-DRA | IFNAR2 | JAK1 | NPLOC4 | PRKCD | SMPD1 | TRIM31 |
| ACTR3 | CCR2 | DHX33 | HLA-DRB1 | IFNB1 | JAK2 | NR1H2 | PRKDC | SNCA | TRIM32 |
| ADAMTS13 | CCR7 | DHX36 | HLA-DRB3 | IFNG | KIF5B | NR1H3 | PRNP | SOCS1 | TRIM34 |
| ADAR | CD14 | DHX58 | HLA-DRB4 | IFNGR1 | KLHL20 | NR1H4 | PSMB8 | SOCS3 | TRIM38 |
| AIF1 | CD160 | DHX9 | HLA-DRB5 | IFNGR2 | KYNU | NUB1 | PTAFR | SP100 | TRIM5 |
| AIM2 | CD2 | DTX4 | HLA-E | IFNL1 | LAMP3 | OAS1 | PTPN1 | SPN | TRIM56 |
| AQP4 | CD226 | EBI3 | HLA-F | IKBKE | LAPTM5 | OAS2 | PTPN11 | STAR | TRIM6 |
| ARG1 | CD244 | EDN1 | HLA-G | IL10 | LGALS7B | OAS3 | PTPN2 | STAT1 | TRIM62 |
| ASS1 | CD274 | EGR1 | HLA-H | IL12A | LGALS9 | OASL | PTPN22 | STAT2 | TRIM68 |
| AXL | CD276 | EIF2AK2 | HMGB1 | IL12B | LGALS9B | OTOP1 | PTPN6 | STAT6 | TRIM8 |
| B2M | CD3E | EP300 | HMGB2 | IL12RB1 | LGALS9C | OTUD5 | PTPRS | STING1 | TTLL12 |
| BCL3 | CD40 | EPRS1 | HMHB1 | IL12RB2 | LILRA4 | PARP14 | PYCARD | STX4 | TXK |
| BST2 | CD44 | F2RL1 | HPX | IL18 | LILRB1 | PARP9 | PYHIN1 | STX8 | TYK2 |
| BTN3A2 | CD47 | FADD | HRAS | IL18R1 | LILRB4 | PCBP2 | RAB20 | STXBP1 | UBA7 |
| C1QBP | CD58 | FASLG | HSP90AA1 | IL1B | LRRFIP1 | PDCD1LG2 | RAB43 | STXBP3 | UBD |
| CACTIN | CD74 | FCGR1A | HSP90AB1 | IL1R1 | LSM14A | PDE12 | RAB7B | STXBP4 | UBE2G2 |
| CALCOCO2 | CD96 | FCGR1B | HSPD1 | IL1RL1 | LTA | PDE4B | RARA | SUMO1 | UBE2K |
| CAMK2A | CDC34 | FLOT1 | HTRA2 | IL2 | MAVS | PDE4D | RASGRP1 | SYK | UBE2L6 |
| CAMK2B | CDC37 | FOXP3 | ICAM1 | IL20RB | MED1 | PGLYRP2 | REL | SYNCRIP | UFD1 |
| CAMK2D | CDC42 | FZD5 | IFI16 | IL21 | MEFV | PGLYRP3 | RELA | TAX1BP1 | USP18 |
| CAMK2G | CDC42EP2 | G3BP1 | IFI27 | IL23A | METTL3 | PIAS1 | RELB | TBK1 | VAMP3 |
| CAPN2 | CDC42EP4 | GAPDH | IFI30 | IL23R | MID1 | PIN1 | RIOK3 | TDGF1 | VCAM1 |
| CASP1 | CEBPG | GAS6 | IFI35 | IL27 | MIR21 | PLCG2 | RIPK2 | TICAM1 | VIM |
| CCL1 | CGAS | GATA3 | IFI6 | IL27RA | MIR24-1 | PLSCR1 | RIPK3 | TIRAP | VPS26B |
| CCL11 | CHUK | GBP1 | IFIH1 | IL33 | MIR26B | PML | RNASEL | TLR2 | VSIR |
| CCL13 | CIITA | GBP2 | IFIT1 | IL36RN | MIR708 | PNPT1 | RNF125 | TLR3 | WAS |
| CCL14 | CITED1 | GBP3 | IFIT1B | ILRUN | MMP12 | POLA1 | RNF135 | TLR4 | WNT5A |
| CCL15 | CLDN1 | GBP4 | IFIT2 | INHA | MNDA | POLR1C | RNF216 | TLR7 | XAF1 |
| CCL16 | CLEC7A | GBP5 | IFIT3 | INHBA | MRC1 | POLR2E | RNF26 | TLR8 | XCL1 |
| CCL17 | CNOT7 | GBP6 | IFITM1 | IP6K2 | MRE11 | POLR2F | RO60 | TLR9 | XCL2 |
| CCL18 | CR1 | GBP7 | IFITM2 | IRAK1 | MT2A | POLR2H | RPL13A | TNF | XRCC5 |
| CCL19 | CRCP | GCH1 | IFITM3 | IRF1 | MUL1 | POLR2K | RPS6KB1 | TNFAIP3 | XRCC6 |
| CCL2 | CREBBP | GPATCH3 | IFNA1 | IRF2 | MX1 | POLR2L | RSAD2 | TNFSF4 | YTHDF2 |
| CCL20 | CRTAM | GSN | IFNA10 | IRF3 | MX2 | POLR3A | SAMHD1 | TOMM70 | YTHDF3 |
| CCL21 | CTNNB1 | HAVCR2 | IFNA13 | IRF4 | MYD88 | POLR3B | SASH3 | TP53 | YY1 |
| CCL22 | CX3CL1 | HCK | IFNA14 | IRF5 | MYO1C | POLR3C | SCGB1A1 | TPR | ZBP1 |
| CCL23 | CXCL16 | HERC5 | IFNA16 | IRF6 | NCAM1 | POLR3D | SCRIB | TRAF3 | ZC3H12A |
| CCL24 | CYLD | HLA-A | IFNA17 | IRF7 | NDUFA13 | POLR3E | SETD2 | TRAF3IP1 | ZCCHC3 |
| CCL25 | CYP27B1 | HLA-B | IFNA2 | IRF8 | NFKB1 | POLR3F | SHFL | TRAIP | ZFPM1 |
| CCL26 | CYRIB | HLA-C | IFNA21 | IRF9 | NFKB2 | POLR3G | SHMT2 | TREX1 | ZP3 |
| CCL3 | DAPK1 | HLA-DPA1 | IFNA4 | IRGC | NLRC3 | POLR3GL | SIRPA | TRIM15 | ZYX |
| CCL3L1 | DAPK3 | HLA-DPB1 | IFNA5 | IRGM | NLRC5 | POLR3H | SLAMF6 | TRIM21 |  |
| CCL3L3 | DCST1 | HLA-DQA1 | IFNA6 | ISG15 | NLRP4 | POLR3K | SLC11A1 | TRIM22 |  |

**Supplementary Table 2: The Univariate and Multivariate cox regression analysis of overall survival in GSE26193**

| Variable | Overall survival | | | |
| --- | --- | --- | --- | --- |
|  | Univariate | | Multivariate | |
|  | HR | *p* - Value | HR | *p* - Value |
| The signature | 0.529 | 0.017 | 0.527 | 0.018 |
| Grade | 1.023 | 0.917 |  |  |
| Stage | 1.842 | <0.001 | 1.824 | <0.001 |

**Supplementary Table 3: The Univariate and Multivariate cox regression analysis of overall survival in GSE51088**

| Variable | Overall survival | | | |
| --- | --- | --- | --- | --- |
|  | Univariate | | Multivariate | |
|  | HR | *p* - Value | HR | *p* - Value |
| The signature | 0.602 | 0.015 | 0.531 | 0.003 |
| Grade | 1.504 | 0.007 | 1.002 | 0.991 |
| Stage | 1.901 | <0.001 | 1.811 | <0.001 |
| Age | 1.032 | <0.001 | 1.039 | <0.001 |

**Supplementary Table 4: DEGs between two groups based on the riskscores of signature**

| FZD5 | MPEG1 | CFD | RCN3 | CDR1 | ADAMDEC1 | OGN |
| --- | --- | --- | --- | --- | --- | --- |
| GCNT3 | SLAMF7 | PTGER2 | BCHE | OMD | TFEC | F13A1 |
| FBXL21 | HS3ST3A1 | VIM | C1QTNF5 | FMO2 | CXCL14 | DCN |
| CA8 | DDR2 | GIMAP8 | MS4A6A | FCGR2B | COL1A2 | MYH11 |
| PDGFRA | PPAPDC1A | RGS1 | SULF2 | LPPR4 | IGSF6 | IGKC |
| SIGLEC1 | FLRT2 | RUNX1 | GPR84 | ENOX1 | ADH1C | IGKV1-5 |
| CYR61 | CYP7B1 | IGF1 | RGS2 | EFEMP1 | TLR8 | LUM |
| HTR2B | CD36 | ADAMTS5 | ALPK2 | ALOX5AP | ACTG2 | COMP |
| LOC441376 | KCNE4 | SELP | GPR18 | CTSK | EDIL3 | COL10A1 |
| PPEF1 | SPP1 | GREM1 | FILIP1L | ZFHX4 | SERPINF1 | THBS2 |
| AIF1 | DEPDC7 | CCL19 | NID2 | C4orf18 | KLRB1 | FAP |
| DKK2 | FIBIN | ABCA6 | EBF3 | HSD17B6 | EBI2 | SFRP2 |
| PTPRC | RASGRP3 | BGN | CD93 | KIAA1913 | FNDC1 | ASPN |

**Supplementary Table 5: Survival analysis of DNA methylated signature-related genes using GSCA database**

| Gene symbol | Tag | Survival type | P-value | HR |
| --- | --- | --- | --- | --- |
| AXL | cg03247049 | OS | 0.9 | 0.89 |
|  | cg03247049 | PFS | 0.84 | 0.84 |
|  | cg03247049 | DSS | 0.9 | 0.89 |
|  | cg03247049 | DFI | 0.62 | 0.55 |
| CCL15 | cg26548883 | OS | 0.58 | 0.60 |
|  | cg26548883 | PFS | 0.74 | 0.74 |
|  | cg26548883 | DSS | 0.58 | 0.60 |
|  | cg26548883 | DFI | 0.19 | 0.00 |
|  | cg23743114 | OS | 0.44 | 0.50 |
|  | cg23743114 | PFS | 0.24 | 0.37 |
|  | cg23743114 | DSS | 0.44 | 0.50 |
|  | cg23743114 | DFI | 0.38 | 0.36 |
| FZD5 | cg13657659 | OS | 0.42 | 2.09 |
|  | cg13657659 | PFS | 0.82 | 1.23 |
|  | cg13657659 | DSS | 0.42 | 2.09 |
|  | cg13657659 | DFI | 0.92 | 1.15 |
| MED1 | cg14430867 | OS | 0.09 | 0.18 |
|  | cg14430867 | PFS | 0.13 | 0.28 |
|  | cg14430867 | DSS | 0.09 | 0.18 |
|  | cg14430867 | DFI | 0.95 | 0.92 |
| POLR3H | cg17554472 | OS | 0.72 | 1.38 |
|  | cg17554472 | PFS | 0.36 | 2.18 |
|  | cg17554472 | DSS | 0.72 | 1.38 |
|  | cg17554472 | DFI | 0.51 | 2.21 |
|  | cg03806693 | OS | 0.37 | 2.28 |
|  | cg03806693 | PFS | 0.57 | 1.66 |
|  | cg03806693 | DSS | 0.37 | 2.28 |
|  | cg03806693 | DFI | 0.92 | 1.15 |
| SLC30A8 | cg23338195 | OS | 0.09 | 0.18 |
|  | cg23338195 | PFS | 0.08 | 0.17 |
|  | cg23338195 | DSS | 0.09 | 0.18 |
|  | cg23338195 | DFI | 0.07 | 0.00 |

**Supplementary Table 6: Prediction of top and bottom 20 drugs targeting positive and negative correlation using CMap database based on signature**

| Drug names | MOA | Gene targets | Perturbation time | Perturbation dose | Replicative correlation  (75^th^ percentile) | Signature strength | TAS | Connectivity score |
| --- | --- | --- | --- | --- | --- | --- | --- | --- |
| HG-5-88-01 | Protein kinase inhibitor | COQ8B\|EGFR | 6 h | 10 uM | 0.21 | 168 | 0.189931 | -0.6875 |
| PFI-1 | Bromodomain inhibitor | BRD4 | 24 h | 3.33 uM | 0.6375 | 214 | 0.372961 | -0.6848 |
| Sorafenib | RAF inhibitor\|FLT3 inhibitor\|KIT inhibitor\|PDGFR inhibitor\|RET inhibitor\|VEGFR inhibitor | RET\|BRAF\|FLT3\|KDR\|RAF1\|FLT1\|FLT4\|KIT\|DDR2\|PDGFRB\|FGFR1\|CYP2B6\|CYP2C8\|CYP3A5\|PDGFB\|SLCO1B3 | 24 h | 10 uM | 0.37 | 322 | 0.349027 | -0.6523 |
| Aminoglutethimide | Glucocorticoid receptor antagonist | CYP19A1\|CYP11A1\|NR3C1 | 24 h | 10 uM | 0.3102 | 82 | 0.161272 | -0.6496 |
| CGK-773 | ATM kinase inhibitor\|ATR kinase inhibitor | ATM\|ATR | 24 h | 10 uM | 0.6 | 330 | 0.449949 | -0.6488 |
| Spironolactone | Mineralocorticoid receptor antagonist | NR3C2\|KCNA5\|KCND3\|SCNN1A\|AR\|CACNA1A\|CACNA1B\|CACNA1C\|CACNA1D\|CACNA1F\|CACNA1G\|CACNA1H\|CACNA1I\|CACNA1S\|CACNA2D1\|CACNA2D2\|CACNA2D3\|CACNB1\|CACNB2\|CACNB3\|CACNB4\|CACNG1\|CYP11B2\|NR3C1\|PGR\|SHBG | 24 h | 20 uM | 0.35 | 260 | 0.305036 | -0.6349 |
| Phenacetin | Cyclooxygenase inhibitor | PTGS1 | 6 h | 10 uM | 0.31 | 72 | 0.151070 | -0.6341 |
| DMBI | PDGFR inhibitor\|VEGFR inhibitor | PDGFRB | 6 h | 10 uM | 0.16 | 113 | 0.135966 | -0.6336 |
| Eflornithine | Ornithine decarboxylase inhibitor | ODC1\|ARG2 | 24 h | 0.04 uM | 0.0288 | 105 | 0.055606 | -0.6297 |
| Tretinoin | Retinoid receptor agonist\|Retinoid receptor ligand | RARG\|RORB\|RARA\|RARB\|ALDH1A1\|ALDH1A2\|GPRC5A\|NR0B1\|NR2C2\|PPARD\|RARRES1\|RORC\|RXRB\|RXRG | 24 h | 0.37 uM | 0.41 | 176 | 0.271631 | -0.6261 |
| Voriconazole | Cytochrome P450 inhibitor | CYP2C19\|CYP51A1\|CYP2C9\|CYP3A4\|CYP3A5\|PTGS1 | 24 h | 0.08 uM | 0.302 | 87 | 0.163906 | -0.6258 |
| BI-2536 | PLK inhibitor | PLK1\|BRD4\|PLK2\|PLK3 | 24 h | 0.04 uM | 0.6783 | 187 | 0.360132 | -0.6244 |
| Atracurium | Acetylcholine receptor antagonist | CHRNA2 | 24 h | 10 uM | 0.35 | 172 | 0.248101 | -0.6225 |
| JNJ-38877605 | MET inhibitor | MET | 24 h | 0.37 uM | 0.6002 | 74 | 0.213105 | -0.6205 |
| Indirubin | CDK inhibitor\|GSK inhibitor | CDK1\|CDK5\|CDK2\|CDK4\|GSK3B\|GSK3A | 24 h | 3.33 uM | 0.38 | 165 | 0.253200 | -0.6189 |
| Betamethasone-acetate | Glucocorticoid receptor agonist | NR3C1 | 6 h | 10 uM | 0.23 | 107 | 0.158630 | -0.617 |
| Phenamil | TRPV antagonist | PKD2L1 | 6 h | 15 uM | 0.48 | 72 | 0.187983 | -0.6129 |
| PNU-282987 | Cholinergic receptor agonist | CHRNA7 | 6 h | 10 uM | 0.19 | 116 | 0.150119 | -0.6114 |
| BRD-K01815685 | Aryl hydrocarbon receptor agonist\|Indoleamine 2,3-dioxygenase inhibitor | AHR\|IDO1 | 24 h | 3.33 uM | 0.1428 | 112 | 0.127880 | -0.6081 |
| Mitotane | Carcinogen | CYP11B1\|CYP11A1\|CYP3A4\|ESR1\|FDX1 | 24 h | 0.37 uM | 0.31 | 101 | 0.178925 | -0.6056 |
| Vinpocetine | Phosphodiesterase inhibitor\|Sodium channel blocker | PDE1A\|SCN10A\|PDE1C | 24 h | 3.33 uM | 0.0393 | 77 | 0.055625 | 0.6053 |
| Cytisine | Acetylcholine receptor agonist | CHRNA4\|CHRNB2\|CHRNA2\|CHRNA3\|CHRNA7\|CHRNA6\|CHRNB4 | 24 h | 10 uM | 0.3168 | 93 | 0.173566 | 0.6053 |
| CPI-1189 | TNF inhibitor | TNF | 24 h | 1.11 uM | 0.2323 | 168 | 0.199761 | 0.6075 |
| ARP-101 | NA | MMP2 | 24 h | 10 uM | 0.18 | 102 | 0.137015 | 0.613 |
| Epirubicin | Topoisomerase inhibitor | TOP2A\|CHD1 | 24 h | 10 uM | 0.8278 | 752 | 0.797815 | 0.6137 |
| Fomepizole | Alcohol dehydrogenase inhibitor | ADH1A\|ADH1B\|ADH1C\|AKR1A1\|CAT | 24 h | 0.125 uM | 0.1248 | 138 | 0.132702 | 0.6156 |
| Ecopipam | Dopamine receptor antagonist | DRD1\|DRD5 | 6 h | 10 uM | 0.1755 | 336 | 0.245549 | 0.6164 |
| Gabazine | GABA receptor antagonist | GABRA1\|GABRA2\|GABRA3\|GABRA4\|GABRA5\|GABRA6\|GABRG2 | 24 h | 3.33 uM | 0.31 | 197 | 0.249888 | 0.6184 |
| SDZ-NKT-343 | Tachykinin antagonist | TACR1 | 24 h | 10 uM | 0.3596 | 206 | 0.275216 | 0.6187 |
| Linopirdine | Potassium channel antagonist | KCNQ2\|KCNQ3\|KCNQ4\|KCNQ5 | 6 h | 10 uM | 0.3 | 194 | 0.243945 | 0.6289 |
| Lomefloxacin | Bacterial DNA Gyrase inhibitor | TOP2A | 24 h | 10 uM | 0.21 | 210 | 0.212349 | 0.6298 |
| Resminostat | HDAC inhibitor | HDAC1\|HDAC2\|HDAC3\|HDAC6\|HDAC8 | 24 h | 3.33 uM | 0.6699 | 497 | 0.583464 | 0.6318 |
| Acivicin | Gamma glutamyltransferase Inhibitors | CAD\|GGT1\|CTPS1 | 24 h | 1.25 uM | 0.1939 | 156 | 0.175866 | 0.6318 |
| MK-2461 | FGFR inhibitor\|VEGFR inhibitor | FGFR1\|FGFR2\|FGFR3\|FLT1\|FLT3\|MET\|FLT4\|KDR\|MERTK\|MST1R\|NTRK1\|NTRK2\|PDGFRB | 24 h | 10 uM | 0.5 | 316 | 0.401938 | 0.6402 |
| OSI-027 | MTOR inhibitor | MTOR | 6 h | 2.22 uM | 0.33 | 155 | 0.228693 | 0.6468 |
| Methylnaltrexone | Opioid receptor antagonist | OPRM1\|OPRK1\|OPRD1 | 24 h | 1.11 uM | 0.0649 | 170 | 0.106213 | 0.6643 |
| Loratadine | Histamine receptor antagonist | HRH1\|CYP2C19\|CYP3A5 | 24 h | 10 uM | 0.1188 | 80 | 0.098579 | 0.6705 |
| Meptazinol | Opioid receptor agonist | BCHE | 24 h | 3.33 uM | 0.3132 | 113 | 0.190231 | 0.6743 |
| Thioridazine | Dopamine receptor antagonist | DRD2\|HTR2A\|DRD1\|HTR2C\|CHRNA7\|MALT1\|ADRA1A\|ADRA1B\|DRD3\|DRD4\|DRD5\|HRH1\|HTR1A\|HTR6\|HTR7\|KCNH2 | 24 h | 10 uM | 0.4373 | 262 | 0.342272 | 0.6777 |
| Etofenamate | Cyclooxygenase inhibitor | PTGS1\|PTGS2 | 24 h | 0.74 uM | 0.364 | 170 | 0.251539 | 0.684 |
